# Monocationic Chlorin as a Promising Photosensitizer for Antitumor and Antimicrobial Photodynamic Therapy

**DOI:** 10.3390/pharmaceutics15010061

**Published:** 2022-12-25

**Authors:** Andrey V. Kustov, Dmitry B. Berezin, Vladimir P. Zorin, Philipp K. Morshnev, Natal’ya V. Kukushkina, Mikhail A. Krestyaninov, Tatyana V. Kustova, Alexander I. Strelnikov, Elena V. Lyalyakina, Tatyana E. Zorina, Olga B. Abramova, Ekaterina A. Kozlovtseva

**Affiliations:** 1 G.A. Krestov Institute of Solution Chemistry, Russian Academy of Sciences (ISC RAS), 153045 Ivanovo, Russia; 2 Institute of Macroheterocyclic Compounds, Ivanovo State University of Chemistry and Technology (ISUCT), 153012 Ivanovo, Russia; 3 Department of Biophysics, Belarussian State University, 220030 Minsk, Belarus; 4 Department of Departmental Surgery and Urology, Ivanovo State Medical Academy (ISMA), 153012 Ivanovo, Russia; 5 Ivanovo Regional Clinical Hospital (IRCH), 153000 Ivanovo, Russia; 6 Laboratory of Experimental Photodynamic Therapy, A.F. Tsyb Medical Radiological Research Center—Branch of the National Medical Research Radiological Center of the Ministry of Health of the Russian Federation, 249031 Obninsk, Russia

**Keywords:** cancer, antibiotic resistant microorganisms, photodynamic therapy, chlorin photosensitizers, synthesis, physicochemical study, antimicrobial and antitumor efficacy

## Abstract

Cancer is one of the leading causes of death worldwide. Despite substantial progress in the understanding of tumor biology, and the appearance of new generations of targeted drugs and treatment techniques, the success achieved in this battle, with some notable exceptions, is still only moderate. Photodynamic therapy (PDT) is a successful but still underestimated therapeutic modality for treating many superficial cancers. In this paper, we focus on the extensive investigation of the monocationic chlorin photosensitizer (PS), considered here as a new photosensitizing agent for both antitumor and antimicrobial PDT. This monocationic chlorin PS (McChl) obtained from methylpheophorbide *a* (MPh) via a two-step procedure is well soluble in water in the physiological temperature range and forms stable complexes with passive carriers. McChl generates singlet oxygen with a good quantum yield in a lipid-like environment and binds mainly to low- and high-density lipoproteins in a vascular system. A comparison of the photodynamic activity of this agent with the activity of the well-established photosensitizer chlorin e_6_ (Chl e_6_) clearly indicates that McChl provides a much more efficient photoinactivation of malignant and microbial cells. The pilot PDT treatment of M1 sarcoma-bearing rats with this PS demonstrates its good potential for further preclinical investigations.

## 1. Introduction

Cancer appears to be one of the major public health problems worldwide, with an incidence rate of twenty million people and a mortality of ten million every year [1,2]. The well-established standard strategies in cancer treatment, i.e., surgery, radiotherapy, chemotherapy and, more recently, immunotherapy and molecular targeted chemotherapy demonstrate promising successes for specific cancers [3,4,5,6]. However, despite this evident progress, the mortality rate and the overall incidence trend in the recent decades remain high [1,2]. This indicates that, to make further progress, it is also necessary to pay attention to other existing but still underappreciated approaches [6,7,8].

PDT is a non-conventional therapeutic modality for treating many superficial and hollow organ tumors [8,9,10,11,12,13,14] as well as localized microbial infections [14,15,16,17,18,19,20]. This easily repeatable light-based technology offers several advantages over the standard treatments, but it still remains underutilized clinically [9,14,20]. The safe and unique efficacy of PDT is its multifunctional ability to cause targeted cancer cell death while being minimally invasive and seldom impeded by drug resistance. It is apparent that the key component of PDT is a photosensitizer that can be accumulated in tumor cells and/or vascular stroma of tumors and is activated by absorbing a photon. This initiates a cascade of photochemical reactions leading to the formation of singlet oxygen (^1^O_2_) and/or other reactive oxygen species (HO^∙^, O_2_^∙–^), which causes cell death (see [9,21,22,23] and refs. therein). The first generation of PSs was based on a porphyrin platform [8,9,14]. Currently, practical applications of these medically approved agents are limited, mainly due to their side effects associated with toxicity and slow elimination from the body, which induces skin photosensitivity persisting for weeks after the tumor treatment [8,9].

The photosensitizers of the second generation, based on natural or synthetic phtalocyanines, chlorins or bacteriochlorins (see [8,14,24,25], have a much higher extinction coefficient in the *Q*-band of the visible spectrum compared to the corresponding extinction coefficient of porphyrins. Among them, chlorin photosensitizing agents are still of special importance to clinical oncology. The most frequently used chlorin PSs are tetra-(*m*-hydroxyphenyl)chlorin (“Foscan”), proposed by R. Bonnett [26,27], mono-*L*-aspartyl chlorin e_6_ (“Talaporfin”) [28] and, especially, chlorin e_6_ trisodium and dimeglumine salts (“Fotoditazin”, “Fotoran e_6_”, “Radachlorin”) [11,29,30,31], developed in the Russian Federation. Most of these PSs are accumulated well by various tumors without the application of specific carriers and quite quickly eliminated from the human body after the tumor treatment. In many instances, they benefit patients entirely in clinical practice. Nevertheless, they do have some disadvantages. Firstly, most chlorin PSs are bound to serum albumin in the circulating blood [32,33,34], although the most effective compounds are delivered by low-density lipoproteins (LDL), providing a receptor-mediated endocytic process by neoplastic cells [8,32,33,34]. Secondly, anionic chlorins, such as ”Talaporfin” or “Radachlorin”, have low affinity towards the lipid membranes of both mammalian and bacterial cells. In particular, this significantly complicates the photodynamic inactivation of Gram-negative pathogens with an outer lipopolysaccharide membrane. Finally, the cost of the multistep synthesis and chromatographic purification of chlorin PSs is often prohibitively high.

The current study focuses on the extensive physical, chemical and biological investigations of the monocationic chlorin photosensitizer, which has the potential to meet some currently unmet PDT needs. The experimental material presented below seems to be a starting point for future preclinical animal studies and provides a comparative analysis of the behavior of McChl and the well-established PS chlorin e_6_.

## 2. Materials and Methods

### 2.1. Photosensitizer Synthesis

**Figure 1:**
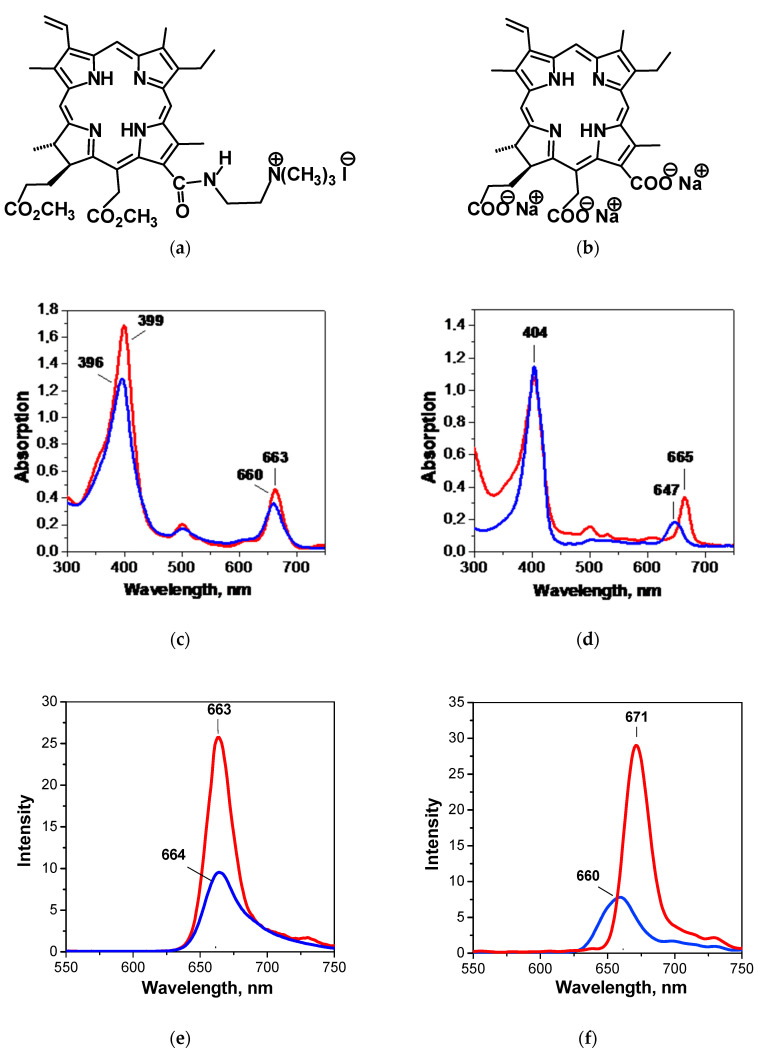
Molecular structures (**a**,**b**), absorption ((**c**,**d**)~1·10^–5^ mol·kg^−1^) and fluorescence ((**e**,**f**);~1·10^–6^ mol·kg^−1^) spectra of monocationic chlorin (**a**,**c**,**e**) and chlorin e_6_ trisodium salt (**b**,**d**,**f**). The blue lines give the values in water, while the red lines refer to liquid 1-octanol (OctOH). The molar extinction coefficients (l cm^−1^ mol^−1^) for the Soret (*B*-) band are 9.098·10^4^ and 9.072·10^4^ (water) and 13.01·10^4^ and 9.955·10^4^ (OctOH) for McChl and Chl e_6_, respectively.

The monocationic chlorin shown in Figure 1 was obtained via the two-step chemical functionalization of MPh described elsewhere [35]. Chlorin e_6_ trisodium salt was purchased from the RANFARMA company (Russian Federation) as a solid powder mixed with polyvinylpyrrolidone (PVP). To obtain pure chlorin e_6_ trisodium salt (see Figure 1), the powder was reprecipitated from cold water at pH ≈ 5.5–6.0, centrifuged, washed with warm water several times to remove residual PVP and centrifuged again. Then, the precipitate was dissolved in a dilute aqueous solution of NaOH at pH ≈ 8.0–8.5 and filtered. After that, the solvent was evaporated in vacuum to obtain solid Chl e_6_ [36]. The final product was identified using ^1^H NMR spectra. A detailed description of the synthesis and identification of the PS is given in the Supplementary Materials File.

### 2.2. Chemicals

Methylpheophorbide *a* was purchased from the “Chlorin” company (Russian Federation) with the purity of 95% [19,35]. The water was distilled twice. Phosphate saline buffer (PSB, Agat-med, for biochemical laboratories) was prepared by dissolving a pure powder in one liter of bidistilled water to reach pH = 7.4. Sodium ethylenediamine tetraacetate (Na_2_H_2_Edta∙2H_2_O, Panreac, chemical pure), glycerol (Gl, Aldrich, 99%), non-ionic surfactant polyoxyethylene (20) sorbitan monooleate (Tween 80, Panreac, pure, pharma grade), dimethylsulfoxide (DMSO, Panreac, > 99%), sodium alginate (Sigma Aldrich, pharmaceutical secondary standard), polyvinylpyrrolidone (PVP, Merck, pharma grade), ε-polylysine *N*~ 30 (ε-Pl, MilkPro, >98%), Thrombin (Renam, pure lyophilisate), calcium chloride (CaCl_2_, Panreac, >99%), “Acrilex P-200” gel (Vekton, pure), RPMI-1640 growth medium (Sigma-Aldrich), fetal calf serum (FCS, Sigma-Aldrich) and human plasma (Ivanovo Regional Transfusion Station, frozen sterile product) were used as supplied.

### 2.3. Physical Chemical Studies

#### 2.3.1. Spectroscopy

The UV-Vis spectra of the PSs were recorded at 293 K with a D8 spectrophotometer (Drawell, China), and the fluorescence spectra were registered with a CM 2203 spectrofluorimeter (Solar, Minsk, Belarus) at 298 K.

#### 2.3.2. Singlet Oxygen Quantum Yield

The quantum yield of singlet oxygen (*Φ*_Δ_) was determined in pure OctOH using both an indirect chemical method and a direct spectroscopic study. A detailed description of both experimental techniques is given in our recent papers [35,36,37].

#### 2.3.3. Solubility

The PS’s solubility was determined by the method of isothermal saturation [38], using spectrophotometry to estimate the solute equilibrium concentration.

#### 2.3.4. Partition Coefficients

The partition coefficients (*P* = *m*_OctOH_/*m*_aq_) between OctOH and phosphate saline buffer (PSB) were also determined by the isothermal saturation method [39]. The equilibrium molality of both PSs was analyzed spectrophotometrically using the previously obtained calibration plots in 1-octanol for McChl and in PSB for Chl e_6_. 

#### 2.3.5. The Interaction with Potential Passive Carriers 

The PS-Tween 80 interaction was studied with the spectrophotometric titration technique described in detail several times [36,40,41]. The PS-PVP interaction was studied in a similar way using the Klotz binding model [42,43]. The experimental data are compiled in the Supplementary Materials File.

#### 2.3.6. Binding to Serum Proteins

The interaction of the PSs with serum proteins was studied using the gel filtration technique [32,44] with a self-built “Acrilex P-200” column (1.5 × 70 cm). Before starting the filtration process, fibrinogen was removed from defrosted plasma by adding pure thrombin and CaCl_2_ at 310 K. After 4 h incubation, pure human serum was accurately separated from the precipitated fibrinogen, divided into ~1.5 mL portions and stored frozen. To start the experiment, one defrosted portion was dissolved in PSB to obtain a 70% serum PSB solution. Then, an appropriate amount of a pigment was dissolved there to obtain a gel filtration medium with an initial PS molality of 80-90 µmol·kg^−1^. This medium was packed into the column above to form a packed bed. After that, the packed bed was equilibrated with PSB as a mobile phase to separate the blood proteins. The mobile phase flow allowed the low-density lipoproteins (LDL) of the plasma to pass almost unhindered through the column, while the smaller high-density lipoproteins (HDL) and, especially, albumin were retarded according to their partial penetration into the gel matrix. Each solution fractions of 2.5 mL were collected and analyzed spectrophotometrically at ~660 nm to obtain the appropriate elution profile (separation curve). The absorption of the serum proteins in PSB was determined at λ = 280 nm. The experimental results are shown in the Supplementary Materials File.

### 2.4. Biological Assays

#### 2.4.1. Photoinactivation of Gram-Negative Antibiotic Resistant Bacteria 

To model antimicrobial PDT, we used several antibiotic resistant nosocomial microorganisms, viz. *Pseudomonas aeruginosa, Enterobacter cloacae*, *Escherichia coli* and *Acinetobacter baumannii*. All the bacteria isolated from human liquids and grown in the Clinical laboratory of IRCH were classified as biosafety level two [15]. This classification means that these microorganisms are capable of causing diseases in humans, but, when standard universal precautions are taken in their handling, they present no health hazard to personnel. The preparation of the liquid bacterial cultures and PS solutions as well as the photoinactivation both in vitro and in vivo were nearly identical to those described elsewhere [19,35], and an appropriate description is given in the Supplementary Materials File.

The study exploiting laboratory animals was performed in agreement with the applicable laws and regulations, clinical practices and ethical principles described in the Declaration of Helsinki. The approval of the Ethical committee of Ivanovo State Medical Academy was obtained (EC 2017.25.10).

#### 2.4.2. Cancer Cellular Uptake and Photoinactivation In Vitro

The cellular uptake and toxicity of the PS in the dark and under irradiation were studied here using K-562 myeloid leukemia cells obtained from the Belorussian Research Center of Pediatric Oncology, Hematology and Immunology (Minsk). All of the manipulations of the cells were identical to those described in our recent papers [36,45]. The PS accumulation was analyzed with a TCS SPE laser scanning confocal fluorescence microscope (Leica, Germany). An argon laser was used as the appropriate excitation source, and the fluorescence emission was registered between 620 and 700 nm.

To analyze the toxicity of the PS under irradiation, K-562 cells were incubated with McChl or Chl e_6_ and then illuminated with red light with an ILM-660-0.5 diode laser (LEMT, Belarus’) for 20–40 s with a light dose between 0.22 and 0.66 J cm^−2^. After irradiation, the cells were incubated again for 3 h. The percentage of dead cells was determined via fluorescence intensity measurements with an FC 500 cytometer and the CXP statistical software package (Beckman Coulter, Brea, CA, USA). The excitation and emission wavelengths were 488 nm and 520 nm, respectively. All the cell experiments mentioned above were repeated at least three times.

#### 2.4.3. In Vivo PDT Modeling

To evaluate the antitumor efficacy of McChl, we performed a pilot PDT study with sarcoma M-1 bearing Wistar rats (a total of 32 animals) at three months of age weighing on average 180–200 g. The animals were purchased from the Biomedical Technology Scientific Center of the Federal Biomedical Agency of Russia (Moscow) and housed in T-4 cages under natural light conditions with forced ventilation of 16 times·h^−1^, at room temperature and with a relative humidity of 50–70%. The rats had free access to water and PK-120-1 food for rodents (Laboratorsnab Ltd., Russia).

The antitumor activity of McChl was studied using a rat sarcoma M-1 model. The tumor strain was obtained from the tumor bank of the N.N. Blokhin National Medical Research Center of Oncology of the Ministry of Health of the Russian Federation. Sarcoma M-1 was implanted subcutaneously as a 1.0 mm^3^ piece of a donor tumor into the outer side of the left thigh. The experiment was started 8–9 days after transplantation, when the largest diameter of a tumor node reached 0.8–1.0 cm. One experimental and three control groups of animals each containing 8 animals were randomly formed, viz. the experimental group (administration of 5 mg·kg^−1^ of McChl followed by PDT with an “Atkus” diode laser (Semiconductor devices, Russia) emitting at 662 nm), and three control groups without irradiation and/or PS administration. The PS was injected intravenously into the tail vein and the treatment was started an hour later. The other details are given elsewhere [46] and in the Supplementary Materials File.

All the animal experiments were carried out according to the Guidelines for the care and use of laboratory animals of the National Medical Research Radiological Centre of the Ministry of Health of Russian Federation and in accordance with the rules and requirements of the European Convention ETS/STE N 123 and the GLP international standard (OECD Guide 1:1998). The animal experimental protocols were approved by the Ethical Committee on the Animal Experiments of the National Medical Research Radiological Centre (N 1-SI-00026).

## 3. Results and Discussion

### 3.1. Physical Chemical Efforts

Figure 1 compares the absorption and fluorescence spectra of McChl and Chl e_6_. We see that the absorption spectra contain the intensive Soret (*B*-) band in the blue light region at λ ≈ 400 nm and the less pronounced *Q*-bands in the green and red light regions between 500 and 670 nm. The most intensive *Q*_x(0-0)_-band at 660 nm, induced by π-π*-electron transfers [47], is within the so-called optical window of tissue [8], providing a good opportunity to realize tumor fluorescence visualization with both macrocycles. The fluorescence spectra reveal a single band between 660 and 671 nm with a moderate Stokes shift. For McChl, the position of the band maximum is almost independent of the solvent nature, while for Chl e_6_ a perceptible hypsochromic shift is observed. The intensity of the fluorescence is found to be more sensitive to the solvent polarity and strongly decreases in aqueous PS solutions.

The quantum yield of singlet oxygen is one of the key parameters of any PS [8,9,14], strongly influencing PDT efficacy. Table 1 compares the corresponding *Φ*_Δ_ values determined in a lipid-like phase. We see that these quantities are high enough and almost identical regardless of the method applied. This indicates that about 60% of the excited PS molecules in a triplet state interact with molecular oxygen, leading to the formation of highly reactive ^1^O_2_. Hence, from the photophysical point of view, the efficacy of monocationic and trianionic chlorins is comparable.

The solubility of any photosensitizing agent in an aqueous medium is of particular importance for clinical utilization. Polyanionic chlorin PSs are well soluble in water or PSB, while insoluble “Foscan” as well as other hydrophobic macrocycles require a carrier [27]. Table 1 shows that the solubility of McChl is high enough for the intravenous administration of 80–100 mg of the PS over a reasonable time. Adding a small amount of PVP increases the solubility by several times, which seems to be important for potential clinical utilization.

The human body is often viewed as a series of lipid-like barriers separating aqueous filled compartments [48], and many of the processes of drug biodistribution depend on the drug’s ability or inability to cross lipid membranes. The partition of the PS between two immiscible phases may give important information about the affinity of a solute towards serum lipoproteins as well as its passive transport through cellular membranes [36]. The most popular model of the inner core of a lipid membrane is liquid 1-octanol (OctOH) [48,49,50], while the water-like pool is often modeled by PSB [36,39].

The partition coefficients given in Table 1 show that both PSs are preferentially accumulated in liquid OctOH. However, McChl reveals more pronounced affinity towards the lipid-like compartment (*p* > 8), with the *P* values being strongly temperature dependent. This feature of McChl behavior leads to the following important findings. First, monocationic McChl should be aggregated in an aqueous solution at lower concentrations than trianionic Chl e_6_. Indeed, our dynamic light scattering and spectrophotometric studies strongly support this finding [36,51,52]. Second, McChl must be accumulated by blood cells more intensively than Chl e_6_ due to its higher affinity towards cell membranes. The results of our previous and recent studies [34,53] also support this prediction. The third and most important feature is that this pronounced affinity of the monocationic chlorin towards the lipid-like compartment must lead to its preferential binding to lipoproteins in the human vascular system. Indeed, our results on gel filtration chromatography, given in Table 1, do indicate that McChl is preferentially bound to LDL and HDL, and that only 10% of the PS is transported by serum albumin. In contrast, about 90% of the Chl e_6_ is bound to serum albumin. Hence, the mechanisms of tumor photoinactivation for these PSs must differ. A significant amount of the Chl e_6_ seems to be deposited in the vascular stroma of tumors, producing marked vascular damage during irradiation. In turn, at least 50% of the McChl can be efficiently accumulated inside tumor cells with variable localization patterns, mostly associated with lipid membranes, to induce necrotic or/and apoptotic cell death. 

Our recent spectroscopic studies [36,40,41,52] have shown that various PSs form stable complexes with the biocompatible non-ionic surfactant Tween 80 [54]. The model parameters, *viz*. lg *K*_b_ and *n*, were recovered from the experimental titration curves by fitting them to Equation (1) [36]:

lg[(*A* − *A*_0_)/(1 − (*A* − *A*_0_))] = lg(*K*_b_) + *n*·lg[*m*_T_^m^ − *n*·*m*_PS_·(*A* − *A*_0_)/(*A*_max_ − *A*_0_)] (1)

where *m*_PS_ is the PS molality equaling ~5–7·10^−6^ mol*·*kg^−1^; *m*_T_^m^ = *m*_T_ − CMC is the molality of aggregated Tween 80 which is evaluated as the difference between its analytical concentration and the critical micellar concentration, CMC = 1.2·10^−5^ M [55]; *n* is the mean number of Tween 80 molecules in close contact with a PS molecule in a micelle; and *A*_0_ and *A*_max_ are the optical densities of fully free (*A*_0_) and fully bound (*A*_max_) PS molecules. 

The experimental optical densities of PS solutions are given in the Supplementary Materials File, and in Table 1 we give the model parameters obtained. Both PSs are found to have two modes of binding with the passive carrier. At low Tween 80 concentrations, they interact with 0.7–0.9 surfactant molecules, indicating a partial incorporation of the PSs into the micelle or the existence of PS-PS contacts. At higher surfactant concentrations, the second mode of PS-carrier binding with the larger lg*K*_b_ and *n* values is realized. We see that both cationic and trianionic chlorins bind strongly to the surfactant micelles, and that Tween 80 may be considered an appropriate passive carrier for the selected photosensitizing agents.

The PS interaction with PVP was studied using the Klotz binding model [43]:

(2) $$\frac{1}{r}=\frac{1}{n}+\frac{1}{n\cdot K_{b}\cdot C_{F}}$$

where *n* is the number of binding sites per mole of a monomer unit and *K*_b_ is the binding constant. The *r* and *C*_F_ values are given by

(3) $$C_{F}=C_{T}\cdot \left(1-\frac{A-A_{0}}{A_{\max}-A_{0}}\right)$$

(4) $$r=C_{T}\cdot \frac{A-A_{0}}{A_{\max}-A_{0}}/C_{\mathrm{PVP}}$$

where *C*_T_ is the total concentration of the PS solution, *C*_F_ is the concentration of the free (unbound) PS and *C*_PVP_ is the polymer concentration. The number of PS binding sites per PVP molecule (*N*_0_) is simply obtained from the *n* values as follows: *N*_0_ = (*M*_PVP_/*M*_PVP mono_) *n*.

Table 1 shows that both charged chlorins form complexes with PVP which are, however, less stable than those they form with Tween 80. One polymer molecule is bound to about 1.5 Chl e_6_ molecules, while for McChl this value is smaller. Similarly, PVP can be considered a convenient passive carrier for stabilizing PS solutions, enhancing the drug solubility and preventing its aggregation at therapeutic concentrations [51].

**Figure 2:**
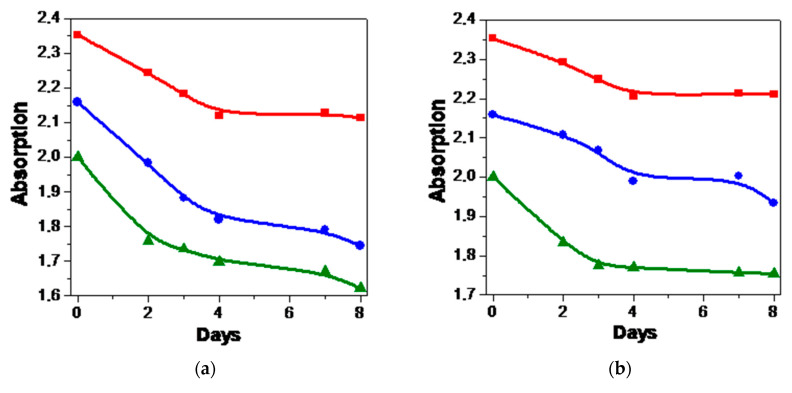

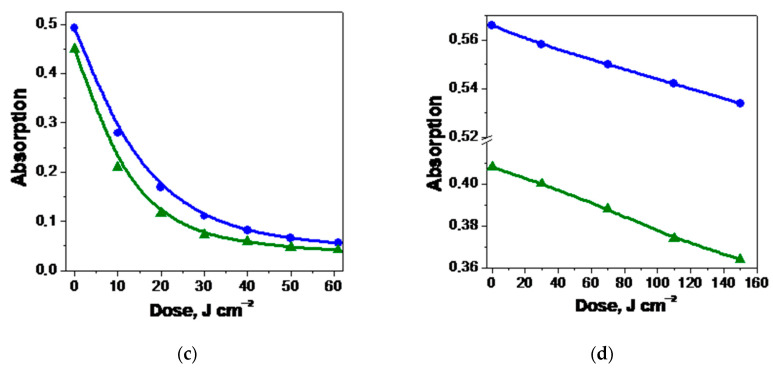
Stability (**a**,**b**) and photostability (**c**,**d**) of PSs in aqueous and non-aqueous media: the dark stability of McChl (▲), Chl e_6_ (●) and Chl e_6_ +PVP (■) in dilute aqueous solutions at 293 K (**a**) and 278 K (**b**); photobleaching of McChl (▲) and Chl e_6_ (●) in water (**c**) and OctOH (**d**) at 293 K. The lines are spline-functions.

The stability of McChl and Chl e_6_ in solutions was examined in the dark and under irradiation using a LED panel (see [19,37] and the Supplementary Materials File). Figure 2a shows that the absorption of PSs in water is reduced by 20% during the follow-up period. We see that either a decrease in temperature or the addition of PVP induces an increase in the dark stability (Figure 2b). However, the effect is quite small, and it is apparent that chlorin PSs should be stored as solids in a cool, dark place, where they remain stable for several years.

The photobleaching study shows that the photostability of McChl and Chl e_6_ in water is quite low, and this solute behavior is very similar to that of other polar chlorin macrocycles. However, in OctOH, both PSs are much more stable, and even a large dose of 150 J·cm^–2^ leads to a 10% decrease in the PS concentration. Taking into account the selective accumulation of McChl in a lipid-like phase (see Table 1), we assume that it has some advantage over more the hydrophilic Chl e_6_.

### 3.2. Antimicrobial PDT

#### 3.2.1. Photoinactivation of Bacteria In Vitro

PDT has been long investigated as an efficient cancer treatment [8,9]. However, it has been gradually recognized that this technology has a powerful antimicrobial effect, especially if the PS applied is cationic [17]. The cationic nature enhances PS binding to microbial cells, which is important for the efficient photoinactivation of Gram-negative pathogens.

Table 2 compares the results of antimicrobial activity towards three antibiotic resistant Gram(-) bacteria belonging to the ESKAPE group [16]. We see that both formulations of Chl e_6_ provide only two logs of killing towards *Pseudomonas aeruginosa*. In contrast, McChl induces the total killing of the pathogen under irradiation. The photodynamic treatment of *Enterobacter cloacae* and *Acinetobacter baumannii* leads to nearly identical results. To enhance the photodynamic activity of Chl e_6_, we used a four-fold higher concentration of the cationic polymer ε-Pl to improve the PS affinity towards the outer membrane of the Gram-negative bacteria. Indeed, this potentiation strongly improves the photodynamic effect. However, the dark toxicity of the PS formulation also increases due to the enhancement of the antimicrobial activity of ε-Pl at higher concentrations.

#### 3.2.2. Photoinactivation of *Pseudomonas aeruginosa* and *Escherichia coli* In Vivo

The modeling of antimicrobial PDT in vivo was performed in a similar way, as recommended by Hamblin et al. [15] and realized in our recent work [19] using laboratory rats with localized burn infections. Here, we examined McChl as a potential candidate for the photodynamic inactivation of pathogens in burn wounds induced by *Pseudomonas aeruginosa* and *Escherichia coli* that are highly resistant to antimicrobial PDT [15]. The PS formulation was identical to the dosage form used before [19] and contained several components that increased the stability of the PS formulation and the PS accumulation (see the footnote to Table 2). The results given in Table 2 show that McChl accomplishes the complete elimination of *Pseudomonas aeruginosa*. However, its killing effect towards *Escherichia coli* is quite moderate and nearly identical to the recent value for the dicationic chlorin containing a myristic acid residue [19]. Apparently, this indicates that either the incubation time was insufficient to induce the efficient binding of the PS to the outer membrane of *Escherichia coli* or the light dose was insufficient. 

Thus, we see that McChl causes fatal damage to Gram-negative bacteria both in vitro and in vivo. The application of potentiating agents such as KI, Na_2_H_2_Edta or ε-Pl [15,16,17,18] may provide additional benefit and reduce the PS concentration and/or the light dose. In contrast, trianionic Chl e_6_ demonstrates a substantial killing effect only at high ε-Pl concentrations.

### 3.3. Antitumor PDT 

#### 3.3.1. PS Accumulation and Photoinactivation of Malignant Cells with McChl and Chl e_6_

It was argued that a PS preferentially binding to LDL was often a better tumor localizer [32,33]. We see from Table 3 that K-562 cells demonstrate three as great an McChl accumulation as Chl e_6_. This value is very similar to the difference in the *p* values given in Table 1 and seems to be responsible for the more efficient photoinactivation of malignant cells accomplished by McChl. Table 3 shows a light dose-dependent killing, and the linear extrapolation predicts the complete elimination of K-562 cells at 2.2 J·cm^−2^. The study of the photoinactivation of HeLa cells leads to similar results, indicating that in vitro McChl is a more efficient PS compared to Chl e_6._

#### 3.3.2. In Vivo PDT Modeling with McChl

To confirm the antitumor efficacy of McChl in vivo, we performed a pilot PDT treatment of sarcoma M1-bearing rats. Table 4 shows that, at an irradiance of 250 mW·cm^−2^ and a light dose of 150 J·cm^−2^, complete tumor regression is observed in all eight of the animals in the PDT group. The treatment induced localized edema accompanied by hyperemia both at the targeted site and in the surrounding soft tissues. This strong acute inflammatory response and necrotic cell death after irradiation may be important to the immune-stimulating function of PDT [8] and in long-term disease control. In contrast, all three of the control groups mentioned above demonstrate continuous tumor growth (see Table 4), indicating that neither McChl nor red light alone are able to kill sarcoma cells. Twenty-one days after the PDT treatment, all of the animals in the PDT group were disease-free (100% tumor inhibition index). However, during the follow-up period of 90 days, two animals (25%) revealed sarcoma recurrence. Thus, the final tumor inhibition index is estimated to be equal to 75%.

## 4. Conclusions

PDT clinical trials are widely known to have been started in the late 1970s. However, this easily repeatable and efficient therapeutic modality is still considered to be a promising antitumor strategy whose potential and appropriate range of applications alone or, better, in combination with other approaches have yet to be shown. Any efficient PDT treatment requires an appropriate photosensitizing agent. It is considered [8,9,14] that the PS must be an individual pure compound with low production costs and good stability in a condensed state. It must have a high absorption peak in the optical window of tissue (between 600 and 820 nm) and a relatively rapid clearance from normal tissues. This photosensitizing agent must be soluble in water or in the aqueous solutions of biocompatible carriers and generate singlet oxygen or other reactive oxygen species with a sufficient quantum yield. However, its most important property is associated with its ability to be accumulated in tumors. The use of the LDL pathway, viz. LDL-bound PSs, is a simple and efficient tool for increasing the selectivity of tumor cell targeting in PDT [32,56]. At first glance, the monocationic chlorin studied here meets all of these requirements. Additionally, this PS demonstrates powerful antimicrobial activity towards Gram-negative bacteria under appropriate irradiation (see Table 3). Our recent study on its acute toxicity towards laboratory rats [57] indicates that the LD_50_ value of this agent is ~100 mg kg^−1^, which is lower than the value for Chl e_6_, which is equal to 180 mg·kg^−1^ [58]. However, this 50% lethal dose for rats is twenty times higher than the PS dose used in the present study (see Table 4). Thus, it seems to be important to continue the analysis of the antitumor efficacy of McChl on various tumor grafts to find the optimal doses and regimes for the treatment. An appropriate pharmacokinetic study dealing with the investigation of PS accumulation and elimination seems to be of particular importance. The combination of McChl with the appropriate passive carriers or with Chl e_6_ may provide additional benefit. The former can improve tumor accumulation, while the latter can enhance the antitumor effect, simultaneously targeting both tumor cells (McChl) and tumor vascular stroma (Chl e_6_).

## Figures and Tables

**Table 1:** Some important physicochemical properties of PSs.

Parameter	McChl			Chl e_6_		
	Singlet oxygen quantum yield, *Φ*_Δ_ [35,37]					
	0.63 ± 0.05 ^1^		0.65 ± 0.07 ^2^	0.56 ± 0.03 ^1^		0.67 ± 0.07 ^2^
	Aqueous solubility and partition coefficient					
*T*, K	Solubility, mg·mL^−1^		*P* [19]	Solubility, mg·mL^−1^		*P* [36]
298.15	1.30 ± 0.10		8.6 ± 0.2	-		1.88 ± 0.06
308.15	1.39 ± 0.09		11.3 ± 0.3			1.90 ± 0.09
318.15	1.22 ± 0.14		14.3 ± 0.3			1.91 ± 0.10
298.15	4.38 ± 0.40 ^3^		-	-		-
	Binding to passive carriers					
	*Tween 80*					
Carrier concentration range, mol·kg^−1 4^	*n*		lg *K*_b_	*n* [36]		lg *K*_b_ [36]
(0.18–1.7)·10^−4^	0.71 ± 0.07		3.73 ± 0.31	0.86 ± 0.01		3.79 ± 0.03
(1.7–6.7)·10^−4^	2.18 ± 0.30		9.36 ± 1.04	1.99 ± 0.10		8.39 ± 0.35
	*PVP, Mw = 10,000 g·mol^−1^*					
	*N*_0_		lg *K*_b_	*N*_0_ [43]		lg *K*_b_ [43]
(0.06–1.1)·10^−4^	0.39 ± 0.11		5.66 ± 0.08	1.62 ± 0.2		4.56 ± 0.09
	Binding to serum proteins					
	LDL	HDL	Albumin	LDL	HDL	Albumin
%	52	39	8	2	6	92

^1^ The values obtained with the indirect chemical method [35]; ^2^ the direct spectroscopic measurements [37]; ^3^ the solubility in a 0.5% aqueous solution of PVP; ^4^ the carrier concentration range refers to McChl. The uncertainties from here on represent the standard error.

**Table 2:** Photoinactivation of Gram (-) antibiotic resistant pathogens in vitro and in vivo.

PS	PS Concentration or Formulation	CFU		
		Dark	40 J·cm^−2^	80 J·cm^−2^
		*Pseudomonas aeruginosa* in vitro ^1^		
McChl	100 µmol·L^−1^	4·10^5^	0	0
	50 µmol·L^−1^	3.1·10^6^	90	0
Chl e_6_	100 µmol·L^−1^	1·10^6^	2.5·10^5^	3·10^4^
	100 µmol·L^−1^ + 0.025% ε-Pl	4·10^5^	1·10^5^	7·10^4^
		*Enterobacter cloacae* in vitro ^1^		
McChl	50 µmol·L^−1^	2·10^5^	0	0
	25 µmol·L^−1^	1.2·10^6^	0	0
Chl e_6_	50 µmol·L^−1^	5.2·10^5^	7.5·10^4^	7·10^4^
	50 µmol·L^−1^ + 0.1% ε-Pl	1·10^4^	60	0
		*Acinetobacter baumannii* in vitro ^1^		
McChl	50 µmol·L^−1^	1.1·10^5^	10	0
Chl e_6_	100 µmol·L^−1^ + 0.1% ε-Pl	1.9·10^5^	60	0
		*Pseudomonas aeruginosa in vivo, wound model*		
McChl	0.25% gel ^2^	4·10^5 3^	-	0
		*Escherichia coli in vivo, wound model*		
McChl	0.25% gel ^2^	2.6·10^5 3^	-	2.5·10^3^

^1^ The sowing dose was 10^7^ CFU and light control without PS gives 6·10^6^, 2·10^6^ and 2·10^6^ CFU for *Pseudomonas aeruginosa, Enterobacter cloacae* and *Acinetobacter baumannii,* respectively; ^2^ the gel formulation contained 0.25% of PS, 10 mass% of Gl, 2 mass% of DMSO, 1 mass% of Tween 80, 0.1 mass% of Na_2_H_2_Edta and 1 mass% of sodium alginate, ^3^ the results of irradiation of the bacteria inoculated onto a wounded skin without the PS.

**Table 3:** PS accumulation and photoinactivation of tumor cells in vitro.

Parameter	McChl	Chl e_6_
	PS accumulation in K-562 cells at *C*_PS_ = 0.2 μM ^1^	
*I*, r.u. ^2^	73 ± 3	23 ± 1
	Cell photoinactivation	
*D*, J·cm^−2^	*Percentage of photodynamically inactivated K-562 cells at C_PS_ = 0.2 μM* ^3^	
0.22	10.3 ± 1.2	7.9 ± 0.9
0.44	21.8 ± 2.1	9.4 ± 1.0
0.66	30.7 ± 2.7	11.2 ± 1.0
	*Survival indexes of HeLa cells at C_PS_ = 1 μM* [34,45] ^4^	
12	3.17 ± 0.04	75.8 ± 3.6

^1^ The concentration of K-562 cells and the incubation time were 10^6^ cells·mL^−1^ and 1 h, respectively; ^2^ the intensity of fluorescence of the absorbed PS at 660 nm; ^3^ the toxicity without light or a PS was found to be within 2%; ^4^ the survival index gives the ratio between the number of living cells in a PS solution and the number of such cells in dark controls, multiplied by 100.

**Table 4:** In vivo PDT modeling with McChl.

Group	Parameter				
	PS dose, mg·kg^−1^		*D*, J·cm^−2^	*P*_s_, mW·cm^−2^	
PDT	5.0		150	250	
Control 1	0		0	0	
Control 2	5.0		0	0	
Control 3	0		150	250	
	Tumor volume, cm^3^				
	*Days*				
	0	7	14	21	90
PDT	0.15 ± 0.02	0	0	0	0 ^2^
Control ^1^	0.15 ± 0.02	13.8 ± 5.3	53.2 ± 13.5	61.3 ± 4.9	-

^1^ The values refer to the first control group; for the other ones, the tumor growth was nearly identical; ^2^ this value refers to the 75% of rats without recurrence.

## Data Availability

The data presented in this study are available on request from the corresponding author.

## Supplementary Materials

The following are available online at https://www.mdpi.com/article/10.3390/pharmaceutics15010061/s1, References [59,60,61,62] are cited in the supplementary materials: S1. Synthesis and identification of chlorin PSs: Scheme S1: Synthesis of monocationic chlorin from methylpheophorbide *a*, Figure S1: ^1^H NMR spectrum of McChl in CDCl_3_, Figure S2: Mass spectrum (MALDI) of McChl, Figure S3: ^1^H NMR spectrum of Chl e_6_ in DMSO d_6_, Table S1: The HPLC study of McChl; S2. PS Stability and photostability studies; S3. The interaction of PSs with potential carriers: Table S2: Spectrophotometric titration of aqueous solutions of McChl and Chl e_6_ by Tween 80 or PVP; S4. PS binding to blood proteins: Figure S4: *B*-splined elution profiles from the “Acrilex P-200” column of PS-doped human serum; S5. Antimicrobial PDT: Figure S5: Toxicity in the dark and under irradiation for a 50 µmol·L^−1^ solution of McChl towards *Enterobacter cloacae* and *Acinetobacter baumannii*, Figure S6: In vivo PDT modelling of a localized burn infection; S6. Antitumor PDT: Figure S7: Accumulation of Chl e_6_ and McChl by K-562 cells.
