# Supplementary material for: Monocationic Chlorin as a Promising Photosensitizer for Antitumor and Antimicrobial Photodynamic Therapy
Source: Pharmaceutics. 2022 Dec 25;15(1):61. doi: 10.3390/pharmaceutics15010061 (PMC9863232; doi:10.3390/pharmaceutics15010061)
Supplement: Supplementary file 1 [file pharmaceutics-15-00061-s001.zip › pharmaceutics-1995956-supplementary.pdf]

Supplementary Material File for

"Monocationic chlorin –promising photosensitizer for antitumor and antimicrobial photodynamic therapy"

by Andrey V. Kustov *et al.*

S1. Synthesis and identification of chlorin PSs

S1.1 Chlorin e6 13(1)-N-(2-N',N',N′-trimethylammonioethyliodide)amide, 15(2),17(3)-dimethyl ester (McChl, comp. 3)

To obtain monocationic chlorin containing one trialkylammonium group (McChl) we performed a simple two-step chemical functionalization of methylpheophorbide *a* (comp. **1**) shown in Scheme S1 [S1]. The discloser of the exocycle of methylpheophorbide *a* with N,N-dimethylethylenediamine (Acros Organics, 99%) was performed in the first stage of functionalization. Then, the intermediate (comp. **2**) was alkylated with methyl iodide (Sigma Aldrich, >99%) to obtain the chlorin required (comp. **3**).

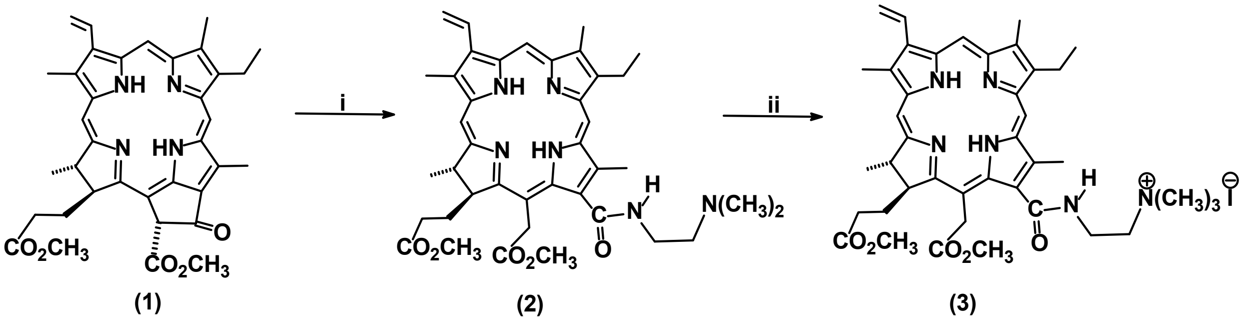

Scheme S1. Synthesis of monocationic chlorin from methylpheophorbide *a*: i: $H_2NCH_2CH_2N(CH_3)_2$, $CHCl_3$, 40 ^{0}C; ii: CH_3I, $CHCl_3$, r.t.

Chlorin e6 13(1)-N-(2-N′,N′-dimethylaminoethyl)amide, 15(2),17(3)-dimethyl ester (comp. ***2****).* 0.082 mmol of pheophorbide *a* 17(3)-methyl ester (comp. **1**) was dissolved in 2 ml of chloroform and 3 mmol of N,N-dimethylethylenediamine was added. The resulting solution was stirred in the dark at 40 ^{0}C under an inert atmosphere for 2.5 h. After washing by water the organic layer was separated and dried with sodium sulfate. Then, the solution was filtered and evaporated to dryness and the final product was carefully purified by silica column chromatography using the chloroform+*iso*-propanol mixture with the volume ratio of 30:1 as an appropriate eluent. The pigment yield was 80%.

Chlorin e6 13(1)-N-(2-N′,N′,N′-trimethylammonioethyliodide)amide, 15(2),17(3)-dimethyl ether (McChl or comp. **3**). 0.043 mmol of chlorin e6 13(1)-N-(2-N',N'-dimethylaminoethyl)amide, 15(2),17(3)-dimethyl ester (comp. **2**) was dissolved in 1.5 ml of chloroform and 16 mmol of methyl iodide was added. The resulting solution was stirred for 1 h at a room temperature. Then, the solvent was evaporated to dryness, and the final product obtained with the quantitative yield was used without further purification.

McChl was identified using ^{1}H NMR-, UV-Vis-, fluorescence emission spectroscopy, MS-spectrometry and, additionally, the HPLC technique combined with mass detection. ^{1}H NMR spectra were registered with a Bruker Avance III spectrometer (500 MHz). $CDCl_3$ was used as an appropriate solvent and TMS as an internal standard. MS-spectra were obtained with a MALDI-TOF MS-spectrometer Shimadzu AXIMA Confidence using a α-cyano-4-hydroxycinnamic acid matrix. UV-Vis spectra were obtained at 293 K with a Drawell G9 spectrophotometer in highly diluted pigment solutions (~10 μmol kg^{-1}). Fluorescence spectra were registered with a CM 2203 spectrofluorimeter (Solar) with the PS concentration of 1 μmol·kg^{-1} at 298 K. The HPLC study

was performed with a Vanquish chromatograph equipped by a Q-exactive HF mass detector (Thermo fisher scientific).

The NMR and MS spectra illustrated in Figures S1, S2 are found to be identical to those given elsewhere [S1-S3]. The results of the HQLC study are shown in Table S1.

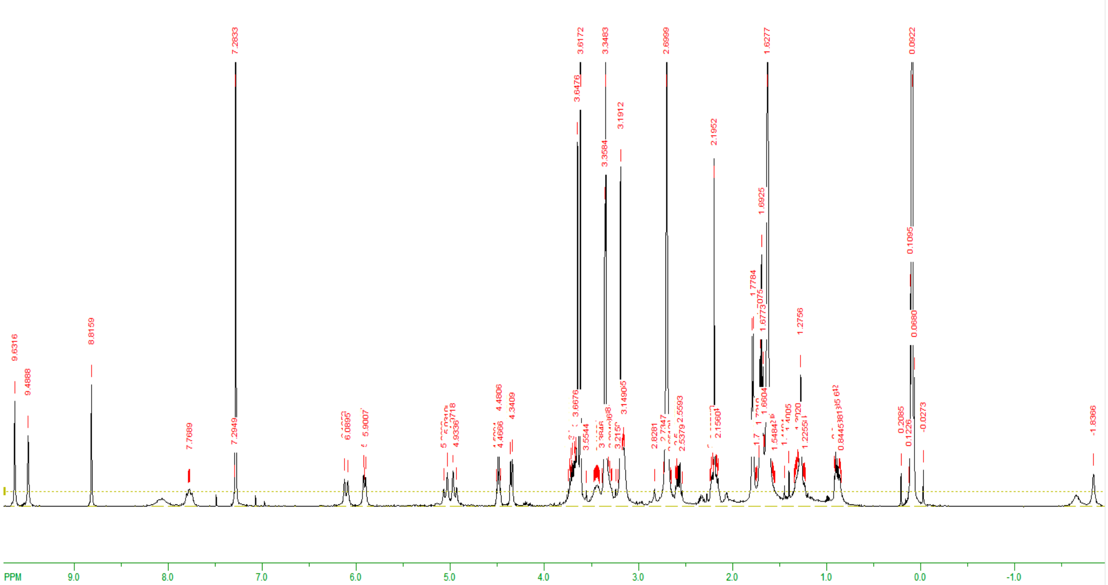

Figure S1. ^{1}H NMR spectrum of McChl in $CDCl_3$.

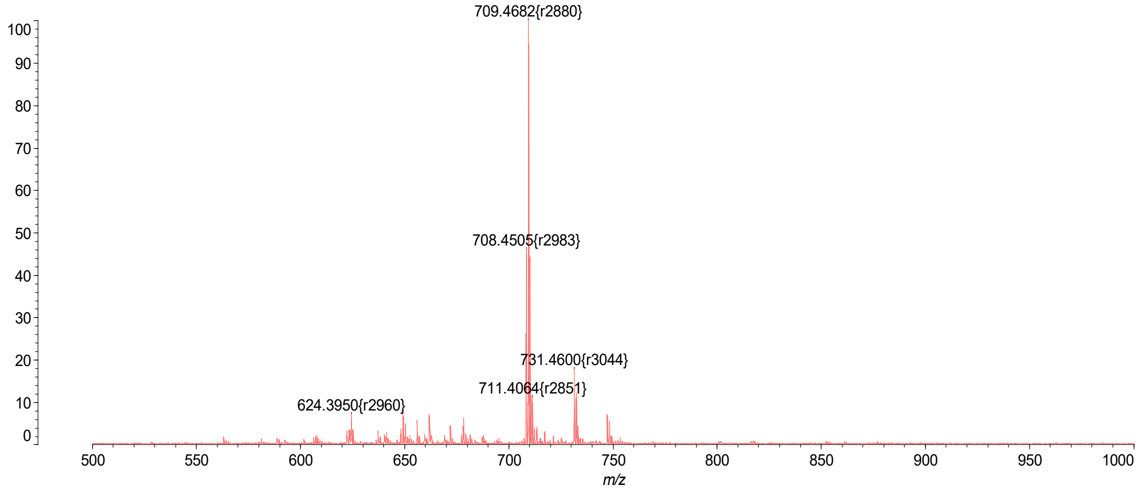

Figure S2. Mass spectrum (MALDI) of McChl.

Mass spectrum: *m/z* (MALDI) (%): *M* = 709.4682 (100), ($[M\text{-}I^-]^+$); 731.4600 (18), ($[M\text{-}I^-\text{+}Na]^+$). Molecular formula $C_{41}H_{53}N_6O_5I$ requires $[M\text{-}I]^+$ 709.4077.

^{1}H NMR spectrum (500 MHz, $CDCl_3$), δ, ppm: 9.54 s (1H, 10-H); 9.46 s (1H, 5-H); 8.79 s (1H, 20-H); 7.72 dd (1H, *J* 17.4 and 11.9 Hz, 3-C<u>H</u>=CH_2); 7.30-7.24 br.m (1H, 13-CON<u>H</u>$CH_2CH_2N(CH_3)_3^+I^-$); 6.05 d (1H, *J* 17.4 Hz, 3-CH=CH<u>H</u> (*trans*)); 5.86 d (1H, *J* 11.9, 3-CH=C<u>H</u>H (*cis*)); 4.93 s (2H, 15-$\underline{CH_2}CO_2CH_3$); 4.49 q (1H, *J* 7.3 Hz, 18-H); 4.34 br.d (1H, *J* 9.2 Hz, 17-H); 3.64 s (6H, 15-$CH_2CO_2\underline{CH_3}$ and 17-$CH_2CH_2CO_2\underline{CH_3}$); 3.58 q (2H, *J* 7.3 Hz, 8-$\underline{CH_2}CH_3$); 3.28 s (6H, 12-CH_3, 7-CH_3); 3.14 s (3H, 2-CH_3); 3.12-2.88 m (4H, 13-CONH$\underline{CH_2CH_2}(CH_3)_3^+I^-$); 2.66 s (9H, 13-CONH$CH_2CH_2N(\underline{CH_3})_3^+I^-$); 17-$\underline{CH_2CH_2}CO_2CH_3$: 2.61-2.53 m (1H) and 2.33-2.14 m (3H); 1.76 d (3H, *J* 7.3 Hz, 18-CH_3); 1.68 t (3H, *J* 7.3 Hz, 8-$CH_2\underline{CH_3}$); -1.55 br.s (1H, 23-NH); -1.80 br.s (1H, 21-NH).

UV/Vis spectra (EtOH) λ_{max} (nm) (log ε): 662 ($Q_{x,(0\text{-}0)}$-, π-π*-, 4.67), 607 ($Q_{x,(0\text{-}1)}$-, π-π*-, 3.66), 529 ($Q_{y,(0\text{-}0)}$-, π-π*-, 3.57), 500 ($Q_{y,(0\text{-}1)}$-, π-π*-, 4.09), 399 (B- (Soret), π-π*-, 5.16). UV/Vis (H_2O) λ_{max} (nm) (log ε): 658 ($Q_{x,(0\text{-}0)}$-, π-π*-, 4.56), 611 ($Q_{x,(0\text{-}1)}$-, π-π*-, 3.76), 528 ($Q_{y,(0\text{-}0)}$-, π-π*-, 3.72), 502 ($Q_{y,(0\text{-}1)}$-, π-π*-, 4.10), 398 (B- (Soret), π-π*-, 5.15). Fluorescence data: λ_{ex} (EtOH) 450 nm, λ_{em} 672 nm, Stokes shift (10 nm or 225 sm^{-1}) [S2].

Table S1. The HPLC study of McChl

Retention time, min.	Square, r.u.	Square, %	Ion	Charge	Empirical formula
11.84	523867474	0.8%	362.2149	2	$C_{39}H_{60}O_7N_6$
13.34	131830431	0.2%	363.20465	2	$C_{41}H_{54}O_6N_6$
14.29	167954066	0.3%	348.1993	2	$C_{40}H_{52}O_5N_6$
15.26	63437351900	**94.9%**	**709.405**	1	$C_{41}H_{53}O_5N_6$
16.06	702528432	1.1%	354.1994	2	$C_{41}H_{52}O_5N_6$
16.5	529969135	**0.8%**	**709.405**	1	$C_{41}H_{53}O_5N_6$
17.08	210538030	0.3%	723.386	1	$C_{41}H_{51}O_6N_6$
17.8	797336194	1.2%	663.3657	1	$C_{39}H_{47}O_4N_6$
23.64	325646819	0.5%	803.54346	1	$C_{46}H_{71}O_6N_6$

Taking into account both components with the molecular weight equal to 709.405, the final purity of McChl is estimated to be of 96 %. This value is comparable with the usual purity of clinically approved chlorin PSs such as "Fotoditazin", "Fotoran e_6" or "Foscan". "Radachlorin" usually contains 70 %-80 % of the main substance and 20-30 % of other chlorins.

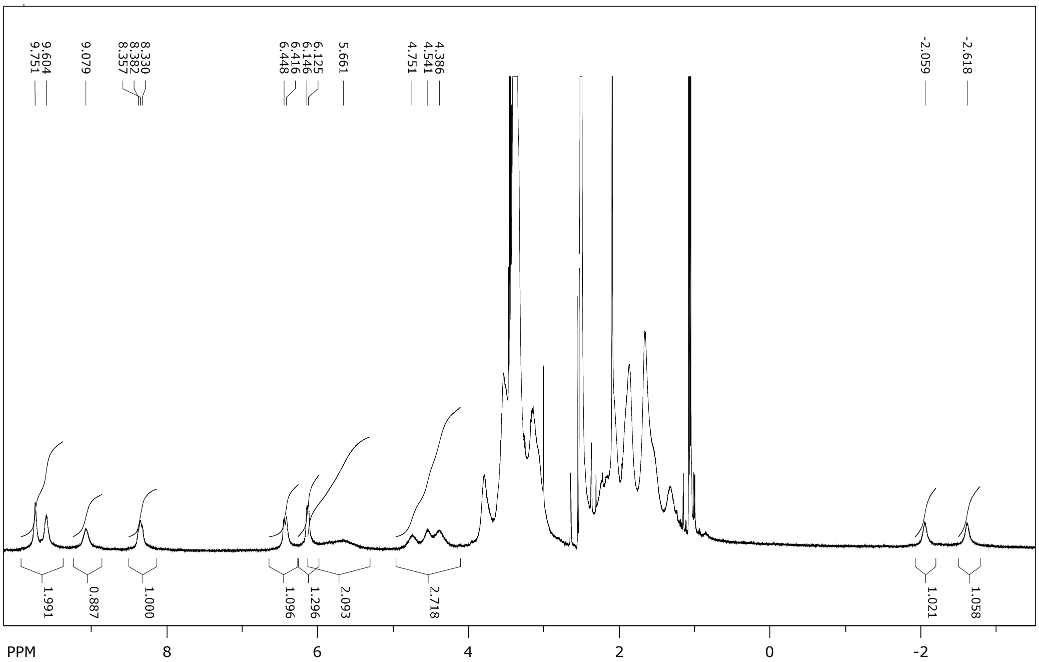

Figure S3. ^1H NMR spectrum of Chl e_6 in DMSO d_6.

S1.2 Chlorin e_6 trisodium salt (Chl e_6, see the main text for the chemical formula) was purchased from the "RANFARMA" company (Russian Federation) as a solid powder mixed with polyvinylpyrrolidone (PVP) and reprecipitated from an aqueous solution to obtain a pure solid form. The purification of the PS from PVP was carried out as follows: 50 mg of Chl e_6+PVP (50/50 per weight) was dissolved in 20 ml of bidistilled water. Then, diluted hydrochloric acid

was added dropwise to achieve pH~5.5-6.0. The precipitate was centrifuged, separated from a liquid phase and washed several times with pure water. The dark-blue powder (18 mg) was dissolved in 20 ml of a dilute aqueous solution of sodium hydroxide (20 ml, pH~8-8.5). The solvent was removed using a rotary evaporator under vacuum and then the final product was carefully dried. The yield of Chl e_6 was 40%. The NMR spectrum is shown in Figure S3.

^{1}H NMR spectrum (500 MHz, DMSO d_6, *J*, Hz), δ, ppm: 9.75 s (1H, 10-H); 9.60 s (1H, 5-H); 9.08 s (1H, 20-H); 8.33 m (1H, 3-C<u>H</u>=CH_2); 6.43 d (1H, *J* 16.0 Hz, 3-CH=CH<u>H</u> (*trans*)); 6.13 d (1H, *J* 10.5 Hz, 3-CH=C<u>H</u>H (*cis*)); 5.66 br. s. (2H, <u>CH_2</u>$COOCH_3$; 4.54 m (2H, 18-H, 17-H); signals from 4 to 1 ppm seem to be covered by residual PVP; -2.06 br.s. (1H, 23-NH); -2.62 br.s. (1H, 21-NH).

S2. Stability and photostability studies

The stability of aqueous PS solutions in the dark at 278±2 K and 293±2 K was monitored spectrophotometrically daily during 8 days. The results are given in Figure 2 a (see the main text). The photostability of both PSs was examined with a 10×10 cm LED panel (BIC, Belarus') emitting between 590 and 720 nm with a maximum at 660 nm. Two quartz cuvettes contained a McChl dilute solution in water or OctOH (~4-5 μmol·kg^{-1}) and an appropriate Chl e_6 solution were irradiated by red light. The intensity of the light spot (power density) was chosen to be equal to ~100 mW·cm^{-2} and was controlled by an "Argus 03" power meter. The results are given in Figure 2 b (see the main text).

S3. The interaction of PSs with potential carriers

The numerical results of titration of McChl or Chl e_6 by Tween 80 and McChl by PVP are given in Table S2.

Table S2. Spectrophotometric titration of aqueous solutions of McChl and Chl e_6 (m_{PS}~·5-7 10^{-6} mol·kg^{-1}) by Tween 80 or PVP

McChl, λ= 665 nm		Chl e_6, λ= 670 nm		McChl, λ= 665 nm	
$m_{Tween\ 80}$·10^4, mol kg^{-1}	*A*, r.u.	$m_{Tween\ 80}$·10^4, mol·kg^{-1}	*A*, r.u.	m_{PVP}·10^4, mol·kg^{-1}	*A*, r.u.
0	0.141	0	0.028	0	0.173
0.0582	0.178	0.229	0.060	0.0614	0.206
0.0848	0.181	0.442	0.078	0.0678	0.225
0.178	0.23	0.667	0.092	0.123	0.216
0.257	0.242	0.88	0.100	0.174	0.24
0.340	0.284	1.08	0.106	0.199	0.256
0.683	0.305	1.30	0.114	0.259	0.258
0.927	0.308	1.53	0.120	0.275	0.274
1.20	0.314	1.76	0.128	0.337	0.277
1.66	0.316	2.78	0.135	0.455	0.267
1.94	0.32	3.63	0.140	0.860	0.291
2.16	0.326	5.44	0.141	1.07	0.301
3.09	0.327	6.27	0.142		
3.97	0.33				
4.88	0.333				
5.79	0.333				
6.70	0.334				

S4. PS binding to blood proteins

The gel filtration technique is an efficient method to separate biomolecules due to the difference in their size [S4]. We used it here to obtain a separation curve by eluting human serum through a column filled by an "Acrilex P-200" gel. The elution profiles obtained (see Figure S4) was similar to those with "Sephacryl S-300" or "Acrilex P-300" gels for similar macrocyclic PSs [S5, S6]. Three protein fractions consisting of low-density lipoproteins (fast-eluting peak), high-density lipoproteins (intermediate peak) and albumins (slow-eluting peak) were detected. We see that both PSs are associated with all the three peaks mentioned above, but in a very different manner. McChl is preferentially bound to LDL and HDL, while Chl e_6 is mainly associated with albumin accounting for about 60 % of the total protein blood content. The amount of PS molecules bound to each type of blood proteins is estimated to be proportional to the squire under the absorption peak of each PS (see Figure S4).

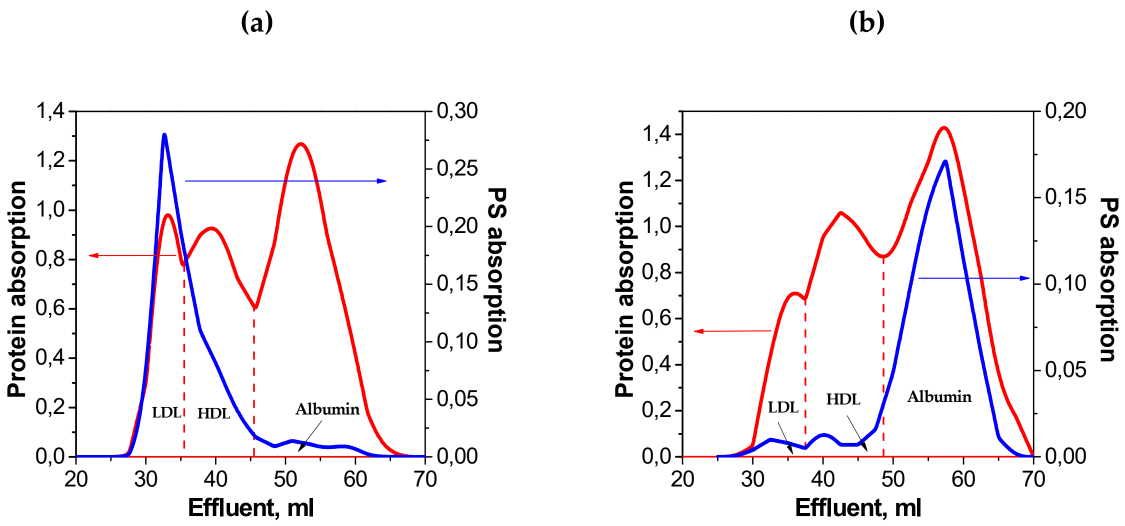

Figure S4. *B*-splined elution profiles from the "Acrilex P-200" column of PS-doped human serum: (a) McChl and (b) Chl e_6. The blue lines refer to the PS absorption profile at 665 nm and the red ones represent the protein absorption at 280 nm.

S5. Antimicrobial PDT

S5.1. Preparation of suspension of microbial cells

Nosocomial antibiotic resistant strains, *viz. Pseudomonas aeruginosa, Escherichia coli, Enterobacter cloacae* and *Acinetobacter baumannii* were isolated from human liquids and accurately grown in the Clinical laboratory of the Ivanovo regional clinical hospital. *Pseudomonas aeruginosa* was found to be resistant towards standard doses of "Meropenem", "Cefepime", "Ciprofloxacin", *etc.*, but sensitive to "Polymixin B". *Enterobacter cloacae* is insensitive towards standard doses of "Furadonin", "Ceftriaxone", "Ciprofloxacin", *etc.*, but sensitive to "Gentamicin sulfate". *Acinetobacter baumannii* is resistant towards "Meropenem", "Imipenem", "Ciprofloxacin", *etc.*, but sensitive to "Polymixin B". *Escherichia coli lactosonegative* was resistant towards "Nevigramon" (Nalidixic acid), "Ciprofloxacin", "Cefamed", "Ertapenem", *etc.* It was moderately sensitive to "Furadonin" or "Gentamicin sulfate".

The daily cultures of the test-strains were grown using microbiological agar mixed with brain-heart infusion broth or the Ol´kenitskiy medium. The cultures were washed by an appropriate amount of saline and diluted to achieve the concentration of $2.4\text{-}2.7 \cdot 10^8$ colony forming units (CFU) per milliliter (0.8-0.9 according to the McFarland standard). The sowing

dose of 10^8 or $2 \cdot 10^7$ CFU ml^{-1} was prepared by a serial dilution of the initial suspension mentioned above.

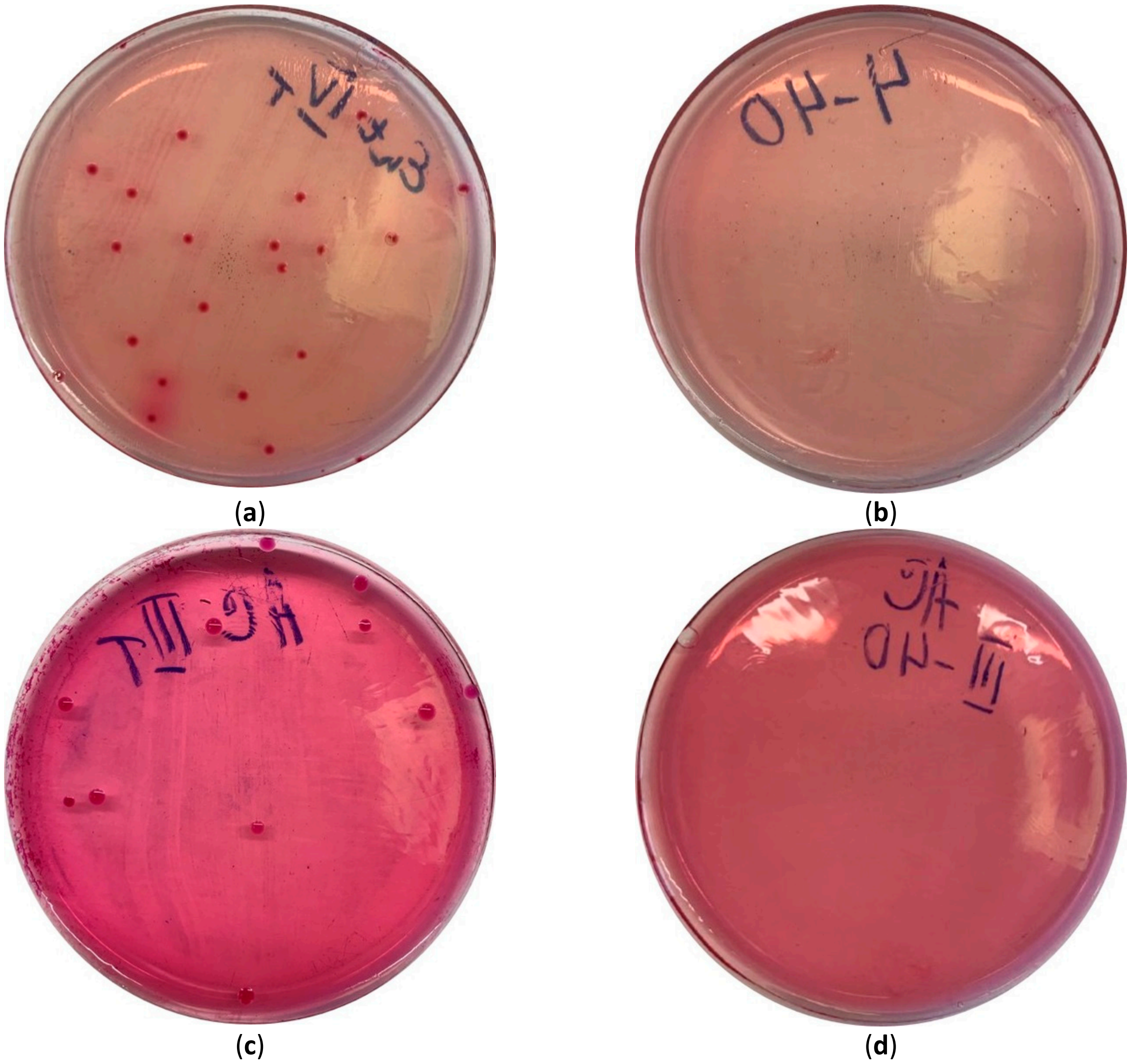

Figure S5. Toxicity in the dark (a, c) and under irradiation (b, d) for a 50 $\mu mol \cdot l^{-1}$ solution of McChl towards *Enterobacter cloacae* (a, b) and *Acinetobacter baumannii* (c, d) (for details see Table 2 in the main text).

S5.2. Photoinactivation of Gram (-) bacteria *in vitro*

Half a milliliter of an aqueous PS solution with an appropriate solute concentration was added to each well of the 4-well plate equipped by a lid. Each well contained 0.5 ml of saline with an appropriate bacterial culture (2 10^7 CFU$\cdot ml^{-1}$). After mixing and incubation in the dark during 20-25 min, the plates were irradiated with a powerful LED panel mentioned above during 7 or 14 min. The total light dose was 40 or 80 J$\cdot cm^{-2}$. The intensity of the light spot (power density) was measured by an "Argus 03" power meter. To model photoinactivation, the three aliquots of microbial cell suspension were prepared. The first aliquot was an original cell suspension (light control); the second one was a suspension with the PS added which was kept in the dark and the third aliquot was a suspension contained the PS to be irradiated with pre-incubation. The second test-culture was incubated at 37 ^{0}C in the stationary incubator during incubation and irradiation of the first and third aliquots (30-45 min). After all manipulations, the test-cultures were mixed and sown with a sterile calibrated loop on petri dishes containing an appropriate

solid growth media (see, for example, photographs in Figure S5). After 24-h incubation, the dishes were counted.

S5.3. Photoinactivation of *Pseudomonas aeruginosa* and *Escherichia coli in vivo*

The PDT study including laboratory animals was performed in agreement with applicable laws and regulations, clinical practices, and ethical principles described in the Declaration of Helsinki. The approval of the Ethic committee of Ivanovo State Medical Academy (ISMA) was provided (EC 2017.25.10).

Female outbred Wistar rats at three-four months of age weighting on average 220-250 g were obtained from the ISMA vivarium and used for an animal model of burn infection. Rats were anesthetized by intramuscular injection of the mixture of Zoletil 100 (Virbac Sante Animale) and Rometar (Bioveta). This procedure was repeated through 2 hours if necessary. After anesthesia, animals were fixed in a prone position using a laboratory stand. Then, the rats were quickly shaved on the back and depilated. Burns were created immediately by applying a self-built round brass electrical heater (d=2 cm) with the temperature of 450 K to the back of each rat during 2-3 seconds to make several non-lethal second-degree burns [S7]. After the burn creation, 0.1-0.15 ml of a sterile saline bacterial suspension (~10^8 CFU·ml^{-1}) was inoculated onto each burn area and spread over its surface.

Twenty minutes after the inoculation of bacteria, McChl was added onto each burn as 0.2 ml of the gel dosage form prepared with the solvent evaporation method. This gel formulation contained 0.25 mass % of the PS, 12 mass % of glycerol, 2 mass % of DMSO, 1 mass % of Tween 80, 0.1 mass % of Na_2H_2Edta and 1 % of sodium alginate was spread over each burn area [S2].

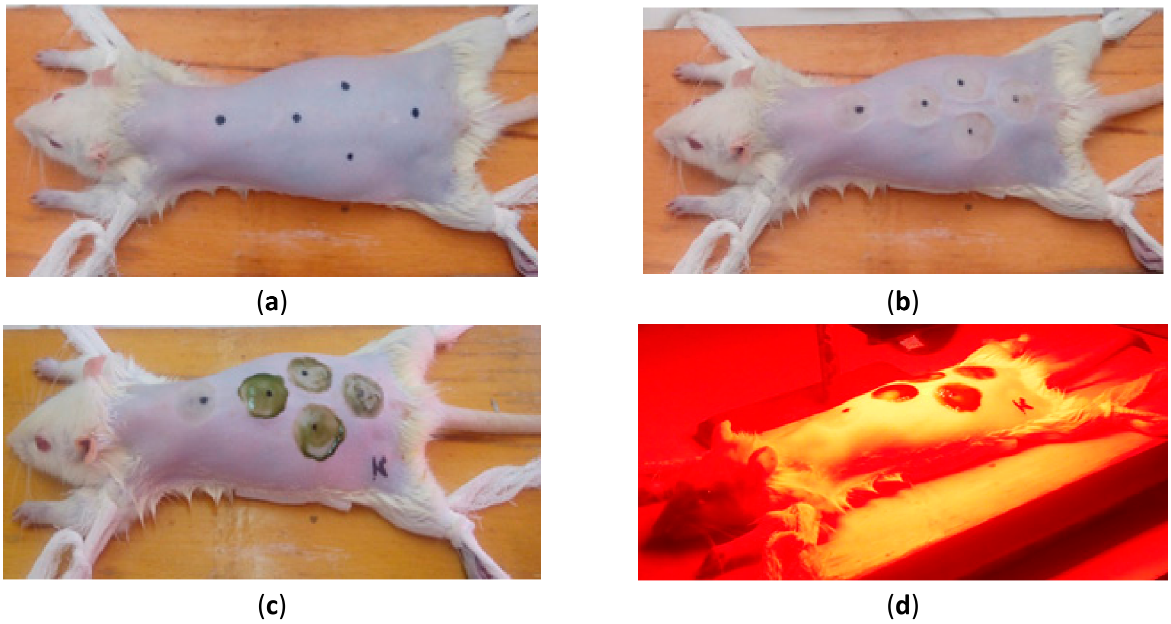

Figure S6. *In vivo* PDT modelling of a localized burn infection: (a) a fixed depilated rat with tagged places to create a burn wound; (b) an animal with inoculated 10^7 CFU of Gram-negative bacteria onto each burn; (c) a rat with inoculated bacteria and added McChl; (d) irradiation of wounds with red light at λ=660 nm and a fluence of 80 J·cm^{-2}. A gel dosage form of the PS was added 20-25 minutes before irradiation.

After further 20 minutes to allow the PS to internalize from the dosage form and bind to bacterial cell membranes, each burn area was illuminated with the LED panel mentioned above. The total light dose was chosen to be equal to 80 J·cm^{-2} delivered during ~14 minutes. The power of light was routinely measured using the power meter above. To estimate the results,

the material from each wound was accurately collected with a sterile cotton swab beginning from the center of each burn area to the periphery, washed by saline and sown with a sterile calibrated loop on petri dishes containing a growth medium according to the Tsarev-Melnikov technique [S8]. After 24-h incubation, the dishes were counted. All animals were alive during the treatment. This work was not designed to allow the rats to recover from anesthesia in an animal warmer and resume their normal activity due to they were capable of causing disease in the vivarium. Thus, they were put to sleep after all manipulations by an additional injection of a lethal dose of anesthetics.

S6. Antitumor PDT

S6.1. PS accumulation and photoinactivation of malignant cells with McChl and Chl e_6

In this paper, PS toxicity in the dark and under irradiation towards malignant cell lines was studied with the K-562 myeloid leukemia cell model and compared with our recent results with the HeLa cell line. The K-562 malignant cells from the collection of the Belorussian Research Center of Pediatric Oncology, Hematology and Immunology (Minsk, Belarus') were cultured in RPMI-1640 growth medium containing 5% v/v of fetal calf serum. Other manipulations were nearly identical to those described before [S9]. In particular, the PSs dissolved in aqueous ethanol were added to RPMI-1640 medium and incubated for 1 h at 37 ^{0}C. Then, an appropriate amount of this mixture was added to a growth medium to reach the final PS concentration of 0.2 or 2 μM and the cell concentration of 10^6 cells·ml^{-1}. After PS accumulation, the cells were washed several times with the growth medium above.

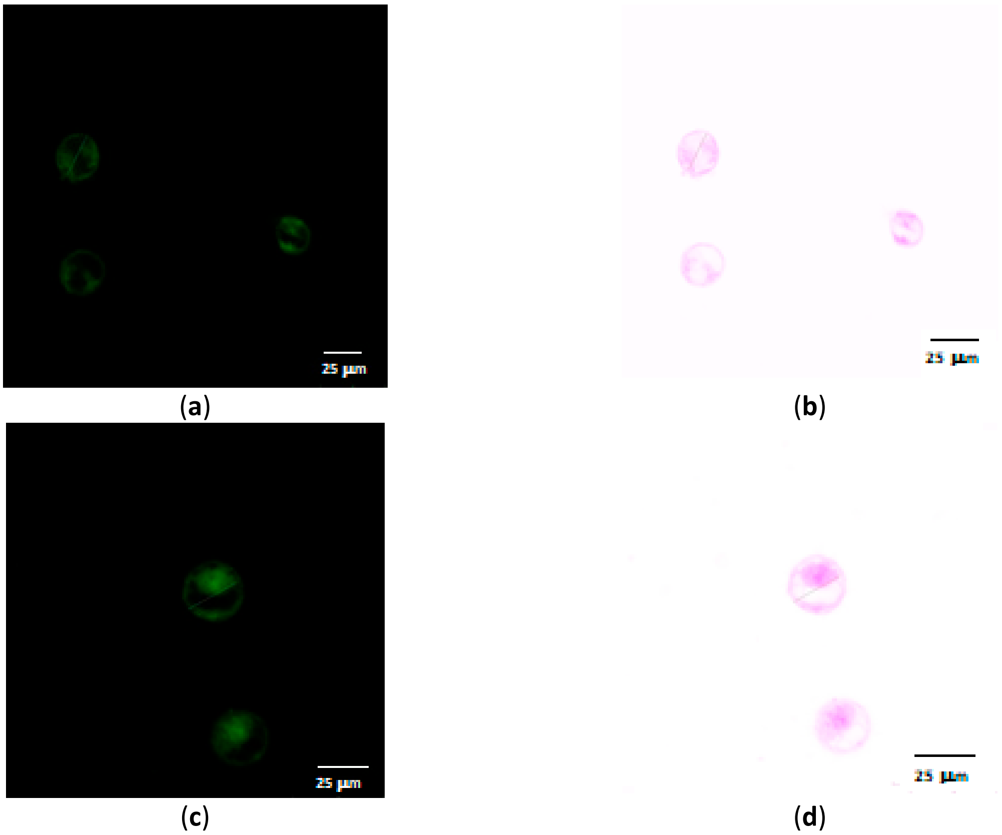

Figure S7. Accumulation of Chl e_6 (a, b) and McChl (c, d) by K-562 cells: (a, c) give the fluorescent images, while (b, d) show the inverted ones. The cell and PS concentration was 10^6 cell·ml^{-1} and 2 10^{-6} mol·l^{-1}, respectively. The incubation time was 2 h and the intensity of PS fluorescence at C_{PS}=2 10^{-6} mol·l^{-1} was 44±4 and 127±6 r.u. for Chl e_6 and McChl, respectively. The scale bars for both cases are 25 μm.

PS accumulation in K-562 cells was studied with a TCS SPE laser scanning confocal fluorescence microscope (Leica, Germany) equipped by an immersion (63x) magnification objective (see Figure S7). An argon laser with λ=488 nm was used as an appropriate fluorescence excitation source. Fluorescence emission was registered from 620 to 700 nm. For the analysis of PS light toxicity, K-562 cells were incubated with an appropriate PS for 1 h and then irradiated by red light with a ILM-660-0.5 diode laser (LEMT, Belarus') for 20 - 40 seconds with the total light dose from 0.22 to 0.66 $J\cdot cm^{-2}$. The power of light was measured with the power meter. After irradiation, the cells were incubated again for 3 h followed by counting. The percentage of dead cells was determined by means of fluorescence intensity measurements with a FC 500 cytofluorometer equipped by a CXP statistical software package (Beckman Coulter, USA). Annexin V was used as an appropriate marker. The excitation and emission wavelengths were 488 nm and 520 nm, respectively. All the cell experiments were repeated 3-4 times.

S6.2. *In vivo* PDT modelling with McChl

This pilot study was performed using sarcoma M-1 bearing Wistar rats (totally 32 animals) at three months of age weighting on average 180-200 g. The animals were purchased from the Biomedical Technology Scientific Center of Federal Biomedical Agency of Russia (Moscow) and housed in T-4 cages under the natural light conditions with the forced ventilation of 16 $times\cdot h^{-1}$, at a room temperature and relative humidity of 50–70%. The rats had free access to water and PK-120-1 feed for rodents (Laboratorsnab Ltd., Moscow, Russia). The antitumor activity of the PS was studied using the M-1 rat sarcoma model. The tumor strain was obtained from the tumor bank of the N.N. Blokhin National Medical Research Center of Oncology of the Ministry of Health of Russian Federation. Sarcoma was implanted subcutaneously as a 1.0 mm^3 piece of donor tumor into the outer side of the left thigh. The experiment was started 8-9 days later, when the largest diameter of the tumor node reached 0.8-1.0 cm. One experimental and three control groups of animals each containing 8 animals were randomly formed, *viz*. the experimental group (administration of 5 mg kg^{-1} of McChl followed PDT with an "Atkus" diode laser (Russia) emitting at λ=662 nm and the power density of 250 $mW\cdot cm^{-2}$ and the total light dose of 150 $J\cdot cm^{-2}$), and three control groups without irradiation and/or PS administration. The PS dose of 5 $mg\cdot kg^{-1}$ for rats corresponds to the human dose of 0.8-0.9 mg kg^{-1} which is normally used in clinical PDT [S10].

Our preliminary fluorescence studies indicated pronounced accumulation of McChl in tumors after 30 minutes of injection and the intensity of PS fluorescence was constant during three hours. Thus, the PS was injected intravenously into the caudal vein and the treatment was started an hour later. The light spot diameter equaled 1.5 cm, the light fluence (total light dose) was 150 $J\cdot cm^{-2}$ and the power density was 250 $mW\cdot cm^{-2}$. After the PDT treatment, all the animals from the experimental groups were returned to cages and kept in the dark during two days to avoid any residual skin phototoxicity.

References:

S1. Gushchina, O.I.; Larkina; E.A., Mironov, A.F. Synthesis of Cationic Derivatives of Chlorin e_6. *Macroheterocycles.* **2014**, *7(4)*, 414-416.

S2. Kustov, A.V.; Belykh, D.V.; Smirnova, N.L.; Venediktov, E.A.; Kudayarova, T.V.; Kruchin, S.O.; Khudyaeva, I.S.; Berezin, D.B. Synthesis and investigation of water-soluble chlorophyll pigments for antimicrobial photodynamic therapy. *Dyes Pigm.* **2018**, *149*, 553-559.

S3. Berezin, D.B.; Kustov, A.V.; Krestyaninov, M.A.; Batov, D.V.; Kukushkina, N.V.; Shukhto, O.V. The behavior of monocationic chlorin in water and aqueous solutions of non-ionic surfactant Tween 80 and potassium iodide. *J. Mol. Liq.* **2019**, *283*, 532-536.

S4. Duong-Ly, K.C.; Gabelli, S.B. Gel filtration chromatography (size exclusion chromatography) of proteins. In: Methods in enzymology. Academic Press, **2014**, *541*, 105-114.
S5. Jori, G. *In vivo* transport and pharmacokinetic behavior of tumor photosensitizers. Ciba Foundation Symposium 146 – Photosensitizing Compounds: Their Chemistry, Biology and Clinical Use. Chichester, UK: John Wiley & Sons, Ltd., **2007**, 78-94.
S6. Khludeev, I.I.; Kozyr', L.A.; Zorina, T.E.; Zorin, V.P. pH-Dependent Changes in the Mechanisms of Transport of Chlorin e_6 and Its Derivatives in the Blood. *Bulletin of Experimental Biology and Medicine*. **2015**, *160(2)*, 208-212.
S7. Huang, L.; Dai, T.; Hamblin, M.R. Antimicrobial photodynamic inactivation and photodynamic therapy for infections. In: Photodynamic Therapy. Methods and Protocols, ed. by Gomer C.J. Humana Press, Totowa: New York, **2010**, 155-173.
S8. Tsarev, V.N.; Nikolaeva, E.N.; Plakhtiy, L.Ya., et al. Oral microbiology, virology and immunology – a textbook. Ed. by Tsarev V.N. Geotar-Media: Moscow, **2013**, 576 p. (in Russian).
S9. Kustov, A.V.; Morshnev, Ph.K.; Kukushkina, N.V. *et al*. Solvation, Cancer Cell Photoinactivation and the Interaction of Chlorin Photosensitizers with a Potential Passive Carrier Non-Ionic Surfactant Tween 80. *Int. J. Mol. Sci*. **2022**, *23*, 5294.
S10 Shekunova, E.V.; Kovaleva, M.A.; Makarova M.N.; Makarov, V.G. Dose selection in preclinical studies: cross-species dose conversion. *The Bulletin of the Scientific Centre for Expert Evaluation of Medicinal Products.* **2020**, 10, 19–28.
